# Minimally Invasive Approaches to Spinal Cerebrospinal Fluid Leak Repair: Current Strategies and a Novel Technique

**DOI:** 10.3390/jpm14111090

**Published:** 2024-11-04

**Authors:** Adham M. Khalafallah, Bhavjeet S. Sanghera, Michael Kader, James V. Boddu, Timur Urakov

**Affiliations:** Department of Neurological Surgery, Miller School of Medicine University of Miami/Jackson Memorial Hospital, Miami, FL 33136, USA

**Keywords:** spine, flexible endoscope, wearable heads-up display, ergonomics, minimally invasive surgery, cerebrospinal fluid leak

## Abstract

Spinal cerebrospinal fluid (CSF) leaks can be caused by tears in the dura and are challenging to treat. Traditional methods of treating spinal CSF leakage include nonsurgical management, epidural blood patches (EBP), and direct surgical repair. Minimally invasive surgery (MIS) is rapidly progressing within neurosurgery due to its advantages for patient safety and comfort. Existing MIS techniques to spine surgery utilize a rigid endoscope, which has limitations when reaching smaller areas requiring greater degrees of visualization. The simultaneous use of a flexible endoscope and wearable heads-up display (wHUD) improves access and visualization in these small areas while allowing the surgeon to maintain optimal ergonomics. In this article, we review minimally invasive approaches to spine surgery and the management of spinal CSF leaks. We also demonstrate a novel minimally invasive technique utilizing flexible endoscopy and a wHUD to treat a case of recurrent CSF leak. We describe the successful utilization of this technology and provide the groundwork for future practitioners to incorporate this approach into their practice.

## 1. Introduction

Although uncommon, cerebrospinal fluid (CSF) leaks are difficult to manage and can be fatal [1]. Traumatic spinal CSF leaks involve a breach of the dura from blunt or penetrating injury that leads to CSF escape from the thecal sac [1,2]. Damage to the dura mater during medical procedures such as spinal taps or laminectomies can precipitate traumatic spinal CSF leaks [3]. Patients can develop intracranial hypotension after the initial breach, which commonly manifests as a positional headache [4].

Conservative management, consisting of antiemetics, hydration, and analgesics, is the cornerstone of initial treatment for spinal CSF leaks [5,6]. Following a course of conservative management, a minimally invasive non-targeted epidural blood patch (EBP) is typically administered [6]. An EBP involves injecting autologous blood into the epidural space [7]. If symptoms persist following multiple EBPs, direct surgical repair is considered [6]. Surgical repair of a CSF leak entails dissection, visualization, and direct suturing or fibrin sealant patch placement to close the defect [2]. While effective, surgical repair is a complex operation associated with greater blood loss and damage to the surrounding tissue [2,8].

In cases where direct localization and repair of CSF leaks is required, endoscopy has been applied to guide instrumentation to the leak site [8]. Traditionally, endoscopic spine surgery has utilized rigid endoscopes, which are set at fixed angles [9]. The tips of flexible endoscopes, by contrast, can rotate, allowing for easier access and visualization along non-linear paths [9].

In this article, we review existing approaches to managing spinal CSF leaks and demonstrate the simultaneous utilization of flexible endoscopy with a wearable heads-up display (wHUD) to treat a case of recurrent spinal CSF leakage. We propose the flexible endoscope as an alternative to rigid endoscopic visualization of the epidural space and discuss applications of wHUDs in spine surgery.

## 2. Minimally Invasive Approaches to Spinal CSF Leak Repair

### 2.1. Epidural Blood Patch

An epidural blood patch is the primary treatment attempted after a period of conservative medical management for patients with CSF leakage [6]. This repair is a minimally invasive method that involves injecting autologous blood into the epidural space through an epidural needle, which can be guided by fluoroscopy [5,7]. In the acute period, it is thought that EBPs improve symptoms by compressing the dural sac to increase pressure [10]. Over time, the EBP occludes the dural tear to halt CSF leakage [10]. While this method can provide acute relief of symptoms, some patients continue to have CSF leaks that require further management [7,11]. In a retrospective review of patients treated with non-targeted EBP, Piechowiak et al. found that 71% of their cohort still had CSF leaks. In their study, they noted that 88% of patients experienced clinical improvement within 1-week follow-up, but only 36% had clinical improvement at extended follow-up [7]. Nevertheless, epidural blood patch repair is a rapid, cost-effective, minimally invasive approach to acutely manage CSF leaks, which remains a strong first-line treatment.

### 2.2. Epidural Fibrin Patch

Fibrin sealant injection is an alternative approach to occluding CSF leaks for patients who do not demonstrate clinical improvement with repeated EBPs [11]. In an epidural fibrin patch, fibrinogen and thrombin are injected to secure tissue and prevent leaks [11,12,13]. To prevent premature fibrin polymerization, this sealant can be administered in two stages: (1) a fibrinogen injection followed by (2) a thrombin solution injection [14]. Compared to EBPs, fibrin sealants have shorter coagulation times and assemble a stronger seal around the dural defect [14]. These sealants have been used as both prophylaxis and treatment for CSF leaks [12].

### 2.3. Targeted Patch Repair

The targeted placement of a patch to repair dural defects can be performed as an MIS treatment for CSF leaks that are refractory to EBPs [15]. Unlike non-targeted EBPs, this technique requires localization of the dural tear. Nonexpandable tubular retractors are used with a microscope to visualize dural defects [15]. Dural tears can be repaired by disconnecting any CSF-venous fistulas and placing a fibrin sealant patch at the defect site [15]. An endoscope can also be used to guide instrument access to the spinal cord for minimally-invasive patch placement [8].

## 3. A Novel Minimally Invasive Approach to Spinal CSF Leak Repair

Minimally invasive surgery (MIS) has become more prevalent in neurosurgery as it minimizes disruption of normal physiology, blood loss, postoperative pain, narcotic use, recovery time, and complications. However, MIS relies upon multiple image-guidance modalities. Adjusting and visualizing these separate images can lead to poor surgical posture, surgeon fatigue, and discomfort [16,17,18]. Wearable heads-up displays solve these issues by allowing the endoscope camera’s field of view (FOV) to be transmitted to the surgeon’s direct line of sight [19]. Our group published the ergonomic advantages of using a wHUD with a rigid endoscope for a lumbar discectomy [20,21]. While useful for a straight “line of sight,” a rigid endoscope has limitations in smaller critical spaces that require more degrees of visualization freedom [22].

Flexible endoscopy can be performed with the use of a wHUD to conveniently display real-time camera output to the surgeon. Endoscopes typically project their FOV to an external monitor in the operating room [23]. This design necessitates that the surgeon shift their view between the patient and the external monitor. The use of a wHUD allows the surgeon to have efficient access to relevant live data without diverting their focus far from the operating table [24]. Improved endoscopic approaches to minimally invasive surgery also reduce the need for fluoroscopic guidance, sparing providers from unnecessary ionizing radiation [25].

While prior studies have reported MIS approaches to CSF leak repair, they have used more invasive surgical repair options [26]. The authors employed a novel MIS approach utilizing a flexible endoscope and wHUD simultaneously to inspect the epidural space in a patient with recurrent spinal CSF leaks that were refractory to multiple EBPs. To our knowledge, this is the first reported spine surgery case using both tools simultaneously in the English literature.

## 4. Illustrative Case

### 4.1. Patient Presentation

**Figure 1:**
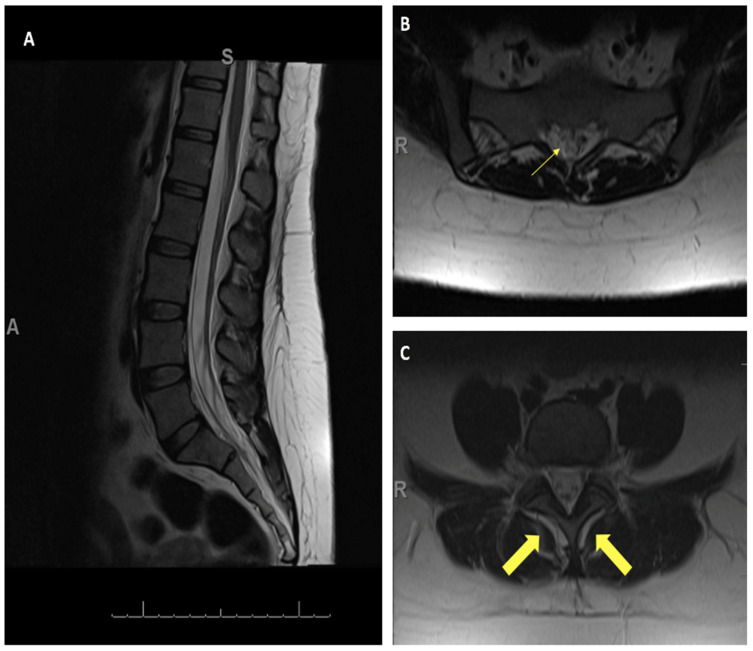
MRI T2 of the lumbar spine. (**A**) Sagittal image (A: Anterior, S: Superior) and (**B**,**C**) axial images showing the area of concern for CSF tracking out of the thecal sac (thin arrow) and CSF signal surrounding the thecal sac and surrounding spinal musculature (thick arrows) (R: Right side).

This is the case of a 31-year-old female with three children delivered via cesarean section. Her third pregnancy required an epidural injection, after which she had persistent positional headaches, requiring multiple epidural blood patches. These provided temporary relief, but her headaches returned. She dealt with these symptoms for over a year before being referred to the neurosurgery department. Due to an iodine allergy, she could not have a CT myelogram; therefore, MRIs of the brain, thoracic, and lumbar spine without contrast were obtained. All were within normal limits, except the MRI of the lumbar spine, which revealed an apparent T2 signal tracking from the dorsal aspect of the thecal sac at lumbar 5 (L5)—sacral 1 (S1) to the surrounding spinal musculature. Additionally, there appeared to be diffuse bilateral fluid collection around the lumbar muscle mass, which did not breach the fascial layer into the overlying fat and skin. (Figure 1)

### 4.2. Novel Surgical Approach

**Figure 2:**
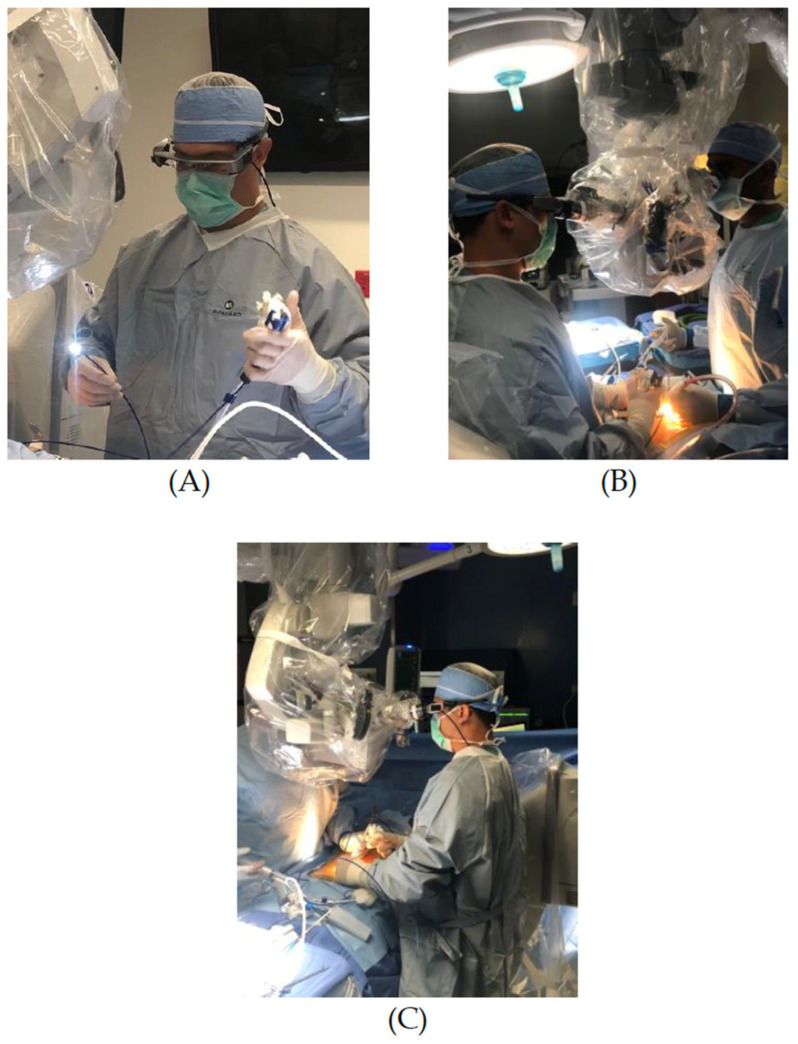
(**A**) Surgeon handling flexible endoscope (SpyGlass by Boston Scientific, Marlborough, MA, USA) while wearing wHUD (Moverio, Epson, Long Beach, CA, USA). (**B**) Use of intraoperative microscope. (**C**) Ergonomic upright front-facing standing position and wHUD.

Given the above findings, a novel approach was considered for treating this patient. The off-label use of a flexible endoscope was submitted to the human subject research office, which determined that it did not need IRB approval. The risks and benefits of the endoscope were explained to the patient, and she consented to the procedure. She was brought into the operating room, positioned prone, and placed under monitored anesthesia care (MAC) sedation. The operating table was flexed to open up the interlaminar spaces. The L5 area was localized with a needle and X-ray. A minimal 3 cm midline incision was made, and right-sided dissection was performed down to the right L5 lamina. A Taylor retractor was used to hold the space open. The interlaminar space of L5-S1 was identified and confirmed with an X-ray. The draped surgical microscope was brought into the field, and a right L5 hemilaminotomy was performed. The ligamentum flavum was opened to evaluate the dura. Microscopic visualization of the dura at this level revealed no obvious openings and defects. At this point, the flexible endoscope (SpyGlass by Boston Scientific, Marlborough, MA, USA) connected with the “plug and play” wHUD (Moverio, Epson, Long Beach, CA, USA) were introduced. With this combination, the surgeon had simultaneous (1) visualization of the endoscopic entry point via the microscope, (2) the flexible endoscopic FOV on their wHUD, (3) control of the endoscope with one hand, and (4) control of the microscope with the other—all from an ergonomic upright front-facing standing position (Figure 2).

**Figure 3:**
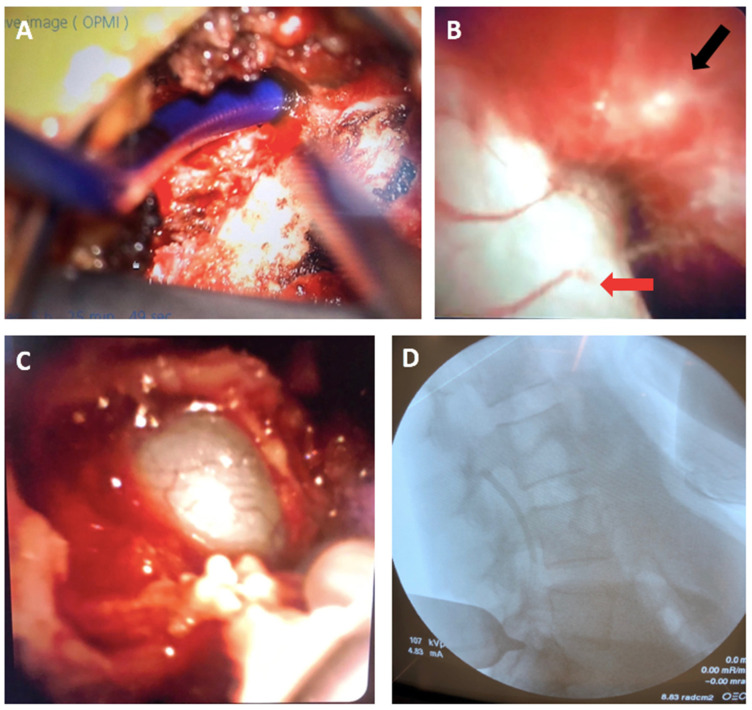
Intraoperative images. (**A**) View from the microscope of the endoscope entering the epidural space at L5-S1 (directed caudally). (**B**) Endoscopic view of the epidural space, with the dura (red arrow) and epidural fat visualized (black arrow). (**C**) Dural defect visualized through the endoscope. (**D**) Lateral fluoro X-ray showing endoscope at L3 level.

Using the flexible endoscope, we were able to navigate to the L3 epidural space and inspect the dura, which had no obvious defects. Around the midline of the L5-S1 area, the endoscope revealed a dural defect as well as an abnormal and paper-thin dura (Figure 3).

The defective and irregular area was covered with a small piece of DuraGen and DuraSeal. The fascia was closed with 0 Vicryl; the skin was closed with 2-0 Vicryl and Monocryl and reinforced with Dermabond. The entire surgical time was 1 h 40 min. The patient went to recovery and was discharged home on the same day.

### 4.3. Postoperative Outcomes

Two weeks post-op, the patient reported an improvement in her headaches and neck pain. Her incision was clean, dry, intact, and flat. Six weeks post-op, she had complete resolution of her preoperative symptoms and remained symptom-free at her 12-month follow-up.

## 5. Flexible Endoscopy in Spine Surgery

Uniportal and biportal endoscopic methods have been applied in spine surgery for lumbar interbody fusions, discectomies, and decompressions [27,28]. Endoscopic spine surgeries usually utilize a rigid endoscope, angled at 0°, 30°, or 45° [9].

In minimally invasive procedures, a high-resolution and wide field-of-view can prevent unnecessary damage to neighboring structures [9]. This is especially important in spine procedures, where surgeons operate adjacent to the spinal cord and nerve roots. However, when attempting to visualize multiple spinal cord levels through a minimally invasive incision, rigid endoscopy is limited by the lordotic and kyphotic curves of the spine [29]. Flexible endoscopy enhances the field-of-view and range-of-motion within a minimally invasive incision, which would be expected to improve endoscopist comfort and reduce the rate of tissue damage.

Yet, applications of flexible endoscopy in spine surgery have been mostly limited to the visualization and removal of cysts, tumors, and adhesions. Zhang et al. demonstrated the feasibility and use of an ultrafine flexible endoscope system to minimize damage to the surrounding nervous tissue when resecting an arachnoid cyst [30]. Similarly, Ren et al. utilized a flexible endoscopic approach to identify a string of schwannomas in the cauda equina and assess the surgical site after resection [31]. Thecaloscopy is a procedure that employs a flexible endoscope to inspect the thecal sac of the spinal cord and cauda equina [32]. This technique has been applied to visualize spinal cysts for removal and perforate adhesions in conditions such as spinal arachnoiditis [29,33]. The ability of flexible endoscopes to examine extensive spinal regions in a minimally invasive procedure has also been applied to detect spinal neurocysticercosis parasites for removal [34].

The flexibility and increased field-of-view that flexible endoscopes provide make them an exciting advancement that may improve patient safety and operator mobility in minimally invasive procedures [9,35]. Currently, there are limited spine-specific flexible endoscopy solutions [9]. Ren et al. used a soft choledochoscope to visualize the cauda equina for a schwannoma resection [31]. Ultrafine flexible endoscopes have small diameters (<1.5 mm), making them well-suited for probing within a narrow, minimally invasive incision to safely examine long spinal cord segments [35]. The development of dedicated flexible endoscopy systems for spine surgery may encourage wider adoption of this endoscopic technique.

## 6. Applications of wHUD with Flexible Endoscopy

Compared to open surgical approaches, endoscopic spine surgery offers the advantages of reducing the damage to tissues surrounding the affected structures, magnification, and shortening postoperative length of stay [27,36].

Traditionally, camera output from the flexible endoscope is projected on an external monitor in the operating room to assist the surgeon in navigation [23]. However, this design has been critiqued for its poor ergonomics, with high rates of musculoskeletal injuries among providers [37]. The monitors used during endoscopic surgery are often difficult to move, making it challenging for surgeons to keep the endoscope and monitor in the optimal ergonomic position, which can lead to discomfort [38]. An assessment of 56 providers performing endoscopies found a 75% incidence of muscle pain and numbness [37].

**Figure 4:**
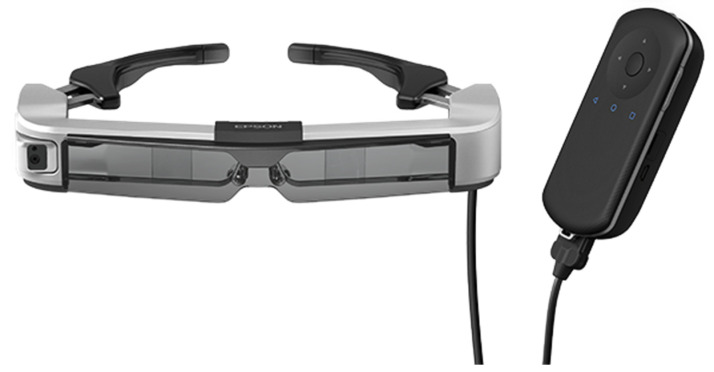
Manufacturer image of a wearable heads-up display (Moverio BT-350, Epson, Long Beach, CA, USA).

Augmented reality (AR) solutions have been implemented in the operating room to provide navigation guidance and data display [19,24]. wHUD-guided flexible endoscopy allows the surgeon to have convenient access to both real-time endoscope FOV and direct view of their environment (Figure 4) [24]. Across specialties, including urology, neurosurgery, craniomaxillofacial surgery, and orthopedic surgery, head-mounted displays have been applied to enhance the surgeon’s field-of-view and improve ergonomics [19,24]. The real-time guidance from AR navigation systems reduces the need for fluoroscopy guidance in minimally invasive procedures, preventing unnecessary radiation exposure [25]. The versatility of flexible endoscopy coupled with the convenience of wHUDs make this technique a promising approach to minimally invasive surgery across all procedures that utilize endoscopy.

## 7. Discussion

Cerebrospinal fluid leaks can occur at the cranial or spinal level. Cranial CSF leaks are most often attributed to traumatic etiologies and may present with rhinorrhea as well as headache [5]. Initial management for cranial CSF leaks can involve conservative medical management or the placement of a lumbar drain [1,5]. Leaks that will not spontaneously resolve with conservative management may require endoscopic or open surgical intervention [1]. Similarly, current methods of managing spinal CSF leaks include conservative medical management, direct surgical repair, as well as minimally invasive epidural blood patches and fibrin injections [3]. Minimally invasive approaches to spine surgery are associated with reduced blood loss, tissue damage, and postoperative length of stay [39].

Ergonomics is of utmost importance for MIS surgeons. Proper ergonomics is associated with longevity, while poor ergonomics is associated with fatigue and musculoskeletal strain/injuries [40,41]. As such, patient, surgeon, and monitor positioning are critical [18]. The wHUD benefits patient care by allowing the surgeon to be in an ideal position—both figuratively and literally. In the past, this tool has been used with long rigid endoscopes [19,20,42,43]. However, flexible endoscopy has yet to be broadly adopted by spine neurosurgeons, let alone in combination with a wHUD.

This report highlights the advantages of using flexible endoscopy with a wHUD for the targeted closure of recurrent CSF leaks. It allowed for (1) a 3 cm opening to visualize a large portion of the lumbar epidural space, (2) different degrees of viewing freedom, and (3) simultaneous views of the microscopic and endoscopic FOVs. These advantages benefit the patient, the surgical resident, and the attending surgeon. The patient experiences the benefits of an MIS approach: decreased operative time, decreased postoperative pain, narcotic use, and recovery time. For the surgical resident, it allows for real-time in vivo anatomic education as well as training in an innovative skill set of the future. For the attending surgeon, it leverages advantages classically associated with a more traditional open surgery—i.e., inspection of multiple lumbar levels—while decreasing the disadvantages inherent to MIS approaches, particularly as they relate to poor ergonomics and their long-term effects.

While the proposed technique offers improved ergonomics through a minimally invasive approach, there are limitations that should be acknowledged. The lack of dedicated flexible endoscopy solutions for spine surgery may present a challenge in the broader adoption of this method [9]. Additionally, short battery life has been described as a limitation of wearable heads-up displays [19]. This may present an issue for operations that require prolonged use of the wHUD. However, as new iterations of wHUD systems are released, battery power would be expected improve. Finally, developing comfort with the operation of a flexible endoscope while simultaneously monitoring feedback on a wHUD may present a learning curve to providers.

Additional research is needed to compare the efficacy of flexible endoscopy-guided CSF leak repair against existing direct repair techniques. Larger prospective cohort studies to assess operating times, blood loss, patient outcomes, and provider ergonomics may provide the foundation for the broader implementation of this approach in managing recurrent spinal CSF leaks. Furthermore, we hope to see applications of the presented technique in the management of other spine conditions to improve provider ergonomics and reduce incision sizes.

## 8. Conclusions

Multiple minimally invasive methods are currently utilized to manage spinal cerebrospinal fluid leaks. While epidural blood patch repair is the primary intervention used to treat CSF leaks, recurrent leaks may require a more targeted direct repair method. Flexible endoscopy with a wHUD is an innovative, safe, and effective tool that can be added to the neurosurgical armamentarium to manage spinal CSF leaks. As with all surgical advances, there is a learning curve associated with gaining a new skill set. However, it is the belief of the authors that using flexible endoscopy can be rapidly learned and employed. Additionally, the presented novel surgical principles can be easily applied across surgical disciplines and can greatly increase resident education in a cutting-edge way.

## Data Availability

The original contributions presented in the study are included in the article, further inquiries can be directed to the corresponding author/s.
